# Practice Implications of Colonoscopic Investigation of Microscopic Colitis in Patients Above 50 Years of Age Presenting With Chronic Diarrhoea: A Multi-Centre Review

**DOI:** 10.7759/cureus.54865

**Published:** 2024-02-25

**Authors:** Audrey Kim, Mary Teoh, Linda Vu, Alfredo Noches-Garcia, Munyaradzi G Nyandoro

**Affiliations:** 1 General Surgery, Fiona Stanley Hospital, Perth, AUS; 2 General and Colorectal Surgery, Fiona Stanley Hospital, Perth, AUS; 3 General Surgery, Rockingham General Hospital, Perth, AUS

**Keywords:** complications, chronic diarrhoea, biopsy, colonoscopy, microcolitis

## Abstract

Background

Patients with an unknown cause for chronic diarrhoea will usually undergo a colonoscopy as part of the investigative work-up, and it is acceptable practice for the patients to undergo random biopsies. The optimum number of biopsies has yet to be established. This study investigated the implications of routine random biopsies for diagnosing microscopic colitis in patients 50 years and older who presented with chronic diarrhoea.

Methodology

A retrospective cohort study of a prospectively maintained internal hospital database across three tertiary teaching hospitals in Perth, Western Australia, on participants >50 years old who presented for an elective colonoscopy to investigate chronic diarrhoea between January 2016 and June 2019. Data was captured from medical records, imaging, colonoscopy, and histopathology reports, and patient follow-up was analysed using SPSS v.29 (IBM Corp., Armonk, NY).

Results

There were 216 patients, with the majority female (67%) and a mean age of 64.6 (SD±9.9). Microscopic colitis was identified in 7.4% (95% CI = 3.9-10.9%). Most positive biopsies (81.3%) were from the left colon. The median number of biopsies per case was seven (IQR=5). The median procedure duration and scope withdrawal time were 23 and eight minutes, respectively. Most of the procedures were done by a consultant (77%). Bowel was adequately prepped in 76.9% of the cases.

Univariate analysis demonstrated that the rate of identification of microcolitis was associated with the number of biopsies taken; microcolitis positivity had a higher mean number of biopsies, 10.8 vs 6.7 (p<0.001).

Key complications were a 30-day readmission rate, seven-day re-presentation with acute colitis, post-procedure bleeding, requiring further imaging or angioembolisation and increased length of stay on readmission.

Conclusion

The prevalence of positive biopsies for microcolitis is low (7.4%). Biopsies during colonoscopy are associated with clinically significant morbidity and health care costs. Most positive biopsies were attained from the left colon. It may be time to standardise practice in investigating microscopic colitis as a cause of chronic diarrhoea in patients > 50 years old.

## Introduction

Microscopic colitis (MC) was first described in the literature by Read et al. [1] as a chronic colonic inflammatory disease with an insidious onset, characterised by enduring, intermittent, watery and non-bloody diarrhoea with essentially normal endoscopic findings. In addition, patients present with various levels of dehydration, electrolyte derangements, abdominal pain, and weight loss; [2] the symptoms have a high relapse rate of 30-60% [3]. MC has a propensity to present in middle-aged and elderly, with an average diagnosis between 50 and 70 years, with a predominance in females [4-6]. The pathophysiology is still poorly understood, with various studies postulating several hypotheses. Proposed mechanisms ranged from autoimmune or immune dysregulation, reaction to luminal antigens from medications of infectious agents and possible genetic predisposition [4-6]. Other risk factors associated with MC include female sex, increasing age, coeliac disease, pre-existing autoimmune disease, medications (proton pump inhibitors and non-steroidal) and smoking [4,7].

There are two main subtypes of MC: lymphocytic colitis (LC) and collagenous colitis (CC) [8,9]. Both have very similar clinical presentations, with the main distinguishing features observed histologically. Lymphocytic colitis contains prominent intraepithelial lymphocytosis, whereby collagenous colitis will have a thick subepithelial collagen banding [2,5,10]. MC is very distinct from typical entities of inflammatory bowel diseases (IBD) as IBD causes macroscopic inflammation that is endoscopically visible, coupled with histological changes of crypts abscesses and architectural distortion, chronic inflammation and granulomas [10,11]. Moreover, IBD peaks during late adolescence or early adulthood, and unlike IBD, MC is not associated with an elevated risk of developing colorectal cancer [10,12]. The primary management goal of MC is to achieve clinical remission, defined as <3 stools/day and no watery stool per week [4]. However, the natural disease progression of MC varies significantly between individuals, with the remission rate ranging from 59-93% in LC and 2-92% in CC [13].

The symptoms for MC are non-specific and overlap those of IBD. Therefore, the current literature suggests performing colonoscopy for patients with chronic watery diarrhoea to thoroughly assess the ileocolonic mucosa and perform biopsies to distinguish between the two types of diseases [14-16]. Macroscopically, the colon in MC usually appears normal or has mild non-specific features like erythema or oedema. Hence, confirmation diagnosis of MC is performed histologically, confirmed with pathological features of mucosal oedema, friability, congestion, exudative lesions and abnormal vascular patterns [17-19]. However, these findings are not uniform throughout the colon. Recent systematic reviews have suggested a minimum of six biopsies should be obtained in total from both the right and left sides of the colon while avoiding the rectum [4,20-24]. The diagnostic yield of MC in random biopsy samples via colonoscopy done in patients with chronic watery diarrhoea is very low, ranging from 1.5-16% [25-30]. Several studies have identified clinical parameters that predict a low likelihood of those presenting with chronic diarrhoea having MC [31-34]. Some studies have suggested that the presence or absence of specific clinical risk factors such as gender, age, and use of certain medications should be used as a screening tool for risk stratification in MC and to avoid the cost of a colonoscopy and biopsy unnecessarily [33-35]. The aim of using these prediction models with high specificity was cost saving. Other studies have proposed the usage of faecal leukocytes, faecal calprotectin or faecal eosinophil-derived proteins as alternative diagnostic markers of MC [2,36,37]. These markers have low specificity and sensitivity for MC from data pooled from small studies.

There is currently a lack of consensus and a paucity of evidence regarding the best practice in the diagnosis of micro-colitis using random colon biopsies in patients presenting with chronic diarrhoea. Moreover, the optimum number and location of biopsy sites still need to be determined. This paper investigates the implications of performing routine random colon biopsies for diagnosing MC in patients aged 50 years and older with chronic diarrhoea. The study was undertaken over multiple South Metropolitan adult teaching hospitals in Western Australia. The primary outcome of this study was to determine the diagnostic positive biopsy rate of MC in random colonic samples; the secondary outcomes were to evaluate procedure-related complications and conduct a cost analysis.

## Materials and methods

Participant population

A retrospective analysis of a prospectively maintained internal hospital database was performed on all adult patients aged 50 years or older who presented for an elective colonoscopy to investigate chronic diarrhoea with a negative stool culture between January 2016 and June 2019 across three teaching hospitals (two were tertiary sites) in Western Australia. Relevant retrospective and prospective data on clinicopathology, medical records, imaging, colonoscopy, histopathology reports, and prospective patient follow-up outcomes were reviewed. All data was entered into a database using Microsoft® Excel for Mac 2020.

Body mass index (BMI) was categorised according to the WHO classification system as normal, overweight, and obese (18-24.9, 25.0 to 29.9 and >30.0 Kg/m2), respectively. MC was defined as chronic watery diarrhoea lasting more than three months. Inclusion criteria were all adult patients > 50 years old having a colonoscopy for chronic diarrhoea, and the exclusion criteria were emergency cases, patients < 50 years old, patients with an active infection or currently on antibiotics and those with a history of inflammatory bowel disease. The Clavien-Dindo (CD) classification was used to categorise the complications. The South Metropolitan Health Service (SMHS) Business activity unit provided the procedural funding and average costing information. 

Statistical analysis 

Baseline characteristics and outcome data for each procedure group were described using mean (± standard deviation), median (IQR) or frequencies/proportions (%), depending on the distribution. The nonparametric Mann-Whitney U test analysed outcomes for continuous unpaired variables. Dichotomous results were compared between groups using χ2 or Fisher’s exact tests with no adjustment for multiple comparisons. The univariate analysis evaluated the relationship between the likelihood of having an MC-positive biopsy and other factors. All analyses were performed using SPSS v. 29 (IBM Corp., Armonk, NY), and a two-tailed p-value of <0.05 was considered statistically significant.

Ethics was obtained through the South Metropolitan Ethics Sub-Committee for Quality Improvement (#27821).

## Results

All patient characteristics

In total, 260 participants who met the inclusion criteria were analysed. Of this population, 145 were females (67%), with a mean age of 64.6 years (SD± 9.9). The median overall length of hospital stay was one day (SD± 0), with a median of four days for readmission hospital length of stay (IQR 4). The median BMI was 27.5 (IQR 7). Most of the colonoscopies were performed by consultants (n=167, 77.3%) with a mean procedure duration of 26.6 minutes (SD± 12.8), and the median number of biopsies per case was seven (IQR 5). Most bowels were well prepped based on the Boston Bowel Preparation Scale (BBPS) with an adequate visual field (n=166, 76.9%). In most colonoscopies, the terminal ileum was visualised and biopsied at 95.8% (n= 207) (Table 1).

**Table 1:** Summary of participant characteristics Values are the number of participants (%) unless otherwise indicated. IQR=interquartile range, SD=standard deviation, n=number; BMI: body mass index, BBP = Boston Bowel Preparation Scale, VAS = visual analogue bowel-prep score ¶ Procedure location: de-identified hospital code

Variable		Number (n), Proportion (%)
Demographic characteristics	Gender: Female n (%)	145 (67.1)
	Age (Years: Mean ± SD)	64.6 ± 9.9
	Weight (Kg: Mean ± SD)	89.8 ± 24.3
	BMI (Number: Median, IQR)	27.5 (7)
	Index Length of Hospital (Days: Median, IQR)	1.0 (0)
	Readmit Length of Hospital (Days: Median, IQR)	4.0 (4)
		Mdn: M
Procedure details	Number of biopsies per case (Total: Median, IQR)	7 (5)
	Procedure duration (Mins: Mean ± SD)	26.6 (12.8)
	Scope withdrawal time (Mins: Mean ± SD)	9.5 (3.4)
		n (%)
Grade of endoscopist	Consultant	167 (77.3)
	Fellow	31 (14.4)
	Trainee registrar	18 (8.3)
BBPS	Minor amount of residual stain	50 (23.1)
	Entire mucosa seen	166 (76.9)
VAS	Fair	50 (23.1)
	Adequate	166 (76.9)
Admission finance type	Public	198 (91.7)
	Private	18 (8.3)
Extend of scope	Scope to terminal ileum	207 (95.8)
	Scope to hepatic flexure	9 (4.2)
Tertiary teaching hospital ^¶^	Hospital A	71 (32.9)
	Hospital B	73 (33.8)
	Hospital C	72 (33.3)

All biopsy characteristics

The majority of the biopsies were taken from multiple colonic sections (n=195, 90.3%), while the remaining were taken almost equally from the right colon (n=8, 3.7%), left colon (n= 7, 3.2%) and rectum (n=6, 2.8%). Only 7.4% of biopsies taken were positive for MC (n=16), specifically lymphocytic collagenous colitis, taken mainly from the left colon (n=13, 81.3%). Five to seven biopsies were commonly taken per case (n=77, 35.6%), followed by eight to 11 biopsies (n=62, 28.7%). Other biopsy-proven diagnosis rate was 13.4% (not MC), with the most common diagnosis being hyperplastic polyps (n=10, 29.4%) (Table 2).

**Table 2:** Summary of biopsy characteristics Values are the number of participants (%) unless otherwise indicated. ‡ Microcolitis: histological diagnosis, § Biopsy groups: Pathwest categorisation funding and billing Boston Bowel Preparation Scale; ||Other: other histological biopsy findings

Variable		Number (n), Proportion (%)
Biopsy Location	Multiple colonic sections	195 (90.3)
	Right colon only	8 (3.7)
	Rectum only	7 (3.2)
	Left colon only	6 (2.8)
Microcolitis positive ^‡^	Lymphocytic collagenase colitis	16 (7.4)
Positive Biopsy location	Left colon	13 (81.3)
	Right colon	3 (18.8)
Biopsy category groups ^§^	Two to four biopsies	54 (25.0)
	Five to seven biopsies	77 (35.6)
	Eight to eleven biopsies	62 (28.7)
	Twelve to seventeen biopsies	23 (10.6)
Other biopsy diagnosis ^||^	No	187 (86.6)
	Yes	29 (13.4)
Other diagnosis specified	Hyperplastic polyps	10 (29.4)
	Melanosis coli	8 (23.5)
	Indeterminate colitis	4 (11.8)
	Low-grade tubular adenoma	4 (11.8)
	Sessile serrated adenoma	3 (8.8)

Procedure activity-based funding

The figures demonstrated that all three tertiary hospitals are performing colonoscopies at a loss, whereby the actual cost per case is higher than the amount of funding provided. The cost for colonoscopy to caecum with or without biopsy for hospital A are $6323.05 and $5478.30, respectively, while funding is only around $4200 (all costings are in Australian dollars). For hospital B, there is a net loss of around $300 for colonoscopy to the caecum and a $500 loss for colonoscopy to the caecum with biopsy. In contrast, for hospital C, it costs $2480 for colonoscopy to the caecum and $2823, inclusive of biopsy with funding of $2285 and $2458, respectively (Table 3). 

**Table 3:** Activity-Based Funding Per – Colonoscopy DRG: diagnostic related group; ICD: International Clinical Diagnosis; Proc: procedure description; Bx: biopsy

Hospital Name	Principal Procedure Code	Principal Procedure Desc	ABF Cost $ Per Case DRG	ABF Fund $ Per Case DRG
Hosp A	Proc.ICD 32084-01	Scope to hepatic flexure & Bx	$12,938.30	$8,663.08
Hosp A	Proc.ICD 32090-01	Scope to caecum & Bx	$5,478.30	$4,257.50
Hosp B	Proc.ICD 32084-01	Scope to hepatic flexure & Bx	$2,560.61	$2,677.25
Hosp B	Proc.ICD 32090-01	Scope to caecum & Bx	$2,953.71	$2,488.69
Hosp C	Proc.ICD 32084-01	Scope to hepatic flexure &Bx	$3,346.63	$3,500.20
Hosp C	Proc.ICD 32090-01	Scope to caecum & Bx	$2,823.32	$2,458.57

The average cost of procedure consumables 

A standard biopsy forceps cost $310, with endoscopy units costing $55.65 per minute. The histology costings show that if more biopsies are taken, the cost becomes more worthwhile, where one biopsy analysis costs $97.15 versus analysing 18 or more, which costs $223.30 (Table 4).

**Table 4:** Average $ Costing Per Case – Colonoscopy

Variable		Average $ Costing Per Case
Endoscopy unit costs per minute		$55.65
Procedure room costs	Endoscopist costs	$25.34
	Anaesthetic costs	$28.41
	Nursing costs	$15.00
	Fixed overhead costs	$10.00
Histology costs	One biopsy analysis	$97.15
	Two to four biopsies analysed	$141.35
	Five to seven biopsies analysed	$180.25
	Eight to eleven biopsies analysed	$194.60
	Twelve to seventeen biopsies analysed	$208.95
	Eighteen or more biopsies analysed	$223.30
	Standard biopsy forceps	$310.00
Hospital admission bed occupation costs		
Ward costs	Nursing costs per hour	$37.00
	Support services costs per hour	$28.00
	Pharmacy	$154.06

Likelihood of positive biopsy and parametric factors

Univariate analysis showed significant associations between BBPS and the likelihood of having an MC-positive biopsy. When the entire mucosa is seen on the endoscope, it is significantly associated with a higher likelihood of achieving a positive biopsy diagnosis of 9.6% versus performing a scope and biopsy on a bowel with minor residual stain, which achieves 0% MC-positive biopsy (p=0.026). In addition, the higher the number of biopsies taken, the greater the likelihood of having a positive MC biopsy for microcolitis, 10.8 vs 6.7 (p<0.001). However, no significant differences were detected in patient baseline characteristics, endoscopist grade, extent of scope and biopsy location (Tables 5-6).

**Table 5:** Univariate analysis results – with the likelihood of having a microcolitis-positive biopsy † as the dependent variable. Values are number of patients (%) unless otherwise indicated. * Pearson Chi-Square analysis, and Fisher’s Exact Test (for cell values <5), and * Bolded denotes significance at p<0.05 † Hospital A/B/C = Three South Metropolitan Health Services – Teaching hospital

Variable (N = 216)		Microcolitis Positive (n=16)		
		(n)	(%)	p-value
Region^†^	Hospital A	6	8.5%	0.400
	Hospital B	3	4.1%	
	Hospital C	7	9.7%	
Gender	Male	3	4.2%	0.275
	Female	13	9.0%	
BMI	Normal weight	4	7.4%	0.946
	Overweight	5	6.7%	
	Obese	7	8.0%	
Grade of endoscopist	Consultant	16	9.6%	0.079
	Fellow	0	0.0%	
	Trainee registrar	0	0.0%	
BBP scale	Minor amount residual stain	0	0.0%	0.026*
	Entire mucosa seen	16	9.6%	
Extend of procedure	Scope to TI	0	0.0%	1.000
	Scope to hepatic flexure	16	7.7%	
Location of biopsy	Single site only	0	0.0%	0.602
	Multiple site biopsy	16	8.2%	
Biopsy subcategory	2 to 4	0	0.0%	<0.001*
	5 to 7	1	1.3%	
	8 to 11	9	14.5%	
	12 to 17	6	26.1%	

**Table 6:** Comparison of continuous characteristics for microcolitis-positive participants Values are the number of participants (%) unless otherwise indicated. Nonparametric independent-samples Mann Whitney U test and * Bolded denotes significance at p<0.05. † BMI – Body Mass Index, ‡ LoHS - Length of hospital stay § Procedure duration – total in minutes

Variable (N=216)	Microcolitis		p-value
	No	Yes	
Age (years)	64.9	61.2	0.077
BMI (nn) ^†^	28.9	29.0	0.871
Weight (Kg)	89.9	88.3	0.853
Initial LoHS (days) ^‡ ^	1.0	1.0	0.483
Scope withdrawal time (mins)	9.7	7.9	0.057
Procedure total duration (mins) ^§^	27.2	19.1	0.005*
Total number of biopsies	6.7	10.8	<0.001*
LoHS representation (days)	3.7	-	-

Summary of all cohort post-procedure complications 

The overall complication rate or cohort morbidity was 10.7% (n=23), of which post-procedure bleeding (both primary and secondary) was the most common morbidity reported (n=13, 6%), followed by prolonged hospital stay on readmission (n=10, 4.6%). Most complications were in the low-order CD category (n=12, 5.6%). A similar complication rate was reported for acute colitis requiring further invasive intervention (n=6, 2.8%) and (n=5, 2.3%), respectively. Other post-procedure outcomes were a 30-day representation of (n=15, 6.9%) and readmission (n=12, 5.6%) (Table 7).

**Table 7:** Summary of post-procedure complications - combined (inpatient & outpatient) Values are the number of participants (%) unless otherwise indicated. † Post-procedure haemorrhage, controlled primarily by resolution clips, secondary with re-scope or embolisation ‡ LoHS – length of hospital stay on readmission post-procedure § Further invasive intervention – re-scope for haemorrhage control or angioembolisation with interventional radiology || Bowel perforation – all post-procedure detected perforations were managed expectantly with antibiotics and bowel rest

Variable (N=216)		Number (Proportion) n (%)
Postoperative outcomes	Any 30-day complication	23 (10.7)
	Required imaging evaluation post-scope	16 (7.4)
	Post-procedure bleeding (primary + secondary) ^†^	13 (6.0)
	Prolonged LoHS on readmission ^‡^	10 (4.6)
	Acute colitis post-procedure	6 (2.8)
	Required further invasive intervention ^§^	5 (2.3)
	Bowel perforation ^||^	2 (0.9)
	30-day representation	15 (6.9)
	30-day readmission	12 (5.6)
	Any 90-day cause of mortality	0 (0.0)
	Clavien-Dindo Classification	
	I	12 (5.6)
	II	6 (2.8)
	III	5 (2.3)

## Discussion

Various studies have shown low diagnostic yield for MC when performing a colonoscopy to investigate chronic watery diarrhoea [25-30]. It is still clinically essential to perform a colonoscopy and biopsy in these cases to rule out other underlying sinister, malignant pathology or inflammatory bowel disease because the cohort for MC is similar to participants presenting with a colon malignancy. The current clinical protocols surrounding targeted colonoscopy and biopsy in specific patient age groups still need to be well-established regarding MC. Most studies looked at a wide range of patient populations ranging from 18 to 80 years old, with variable biopsies taken from different biopsy locations. There is currently no consensus on performing routine random colon biopsies for diagnosing MC in patients 50 years and older who present with chronic watery diarrhoea. Moreover, the cost implication of performing such random colonic biopsies has yet to be defined. To our knowledge, this is the first study looking at the impact of performing random colonic biopsies in patients aged 50 years and older.

Incidence and diagnostic yield

The recent literature shows an incidence rate of 4.14 per 100,000 for collagenous colitis and 4.85 per 100,000 for lymphocytic colitis [5]. There is an overall predominance in females, notably for collagenous colitis. The prevalence of MC is seen in older populations; a multicentre review by Tontini et al. reported a prevalence rate of 19% and a five-fold greater risk in those older than 40. Furthermore, the prevalence peaked at its highest in the octogenarians (80 years or older) at 43%, with an average of one MC-positive biopsy of every other colonoscopy observed [28]. A study by Macaigne et al. in France supported the findings that patients aged >50 years old had a higher prevalence of MC. The data suggests that random biopsies when identifying MC should be targeted in patients aged 50 years and older to both lower healthcare costs and negate unnecessary morbidity [35].

Our study demonstrated a significantly low diagnostic yield of 7.4% when performing random colonic biopsies, consistent with several published studies. A review done by Pardi et al. showed a similar yield of 8% with a high proportion in the elderly population with a female predominance, especially with collagenous colitis [5]. This result is congruent with our findings, although we did not find a significant association with gender, as did Macaigne et al. [35]. A meta-analysis by Loganathan et al. [38] of 21 studies also demonstrated a low MC-positive biopsy rate of only 15%, similar to a large NHS study yielding a 5% MC-positive rate [30]. Therefore, this irrefutably demonstrates that random colonic biopsies have low diagnostic yields. 

Costings

The data from our study demonstrated significant costs associated with colonoscopy and biopsy, with all tertiary hospitals in Western Australia operating at a loss. Colonoscopy costs an average of $3000 due to the high per-minute cost of endoscopy suite usage, biopsy equipment and histological analysis. Similar cost findings were noted in various studies in other countries. A retrospective analysis of diagnostic colonoscopies in the United Kingdom reported a 30% biopsy rate, a cost of £26,700 attributed to biopsies, and a low diagnostic yield of 1.5% [29].

Furthermore, Hotouras et al. [39] reported an overall MC incidence of 1.3%, and their cost-analysis noted that an extra £22,057 was spent to diagnose two cases of MC, both of which had spontaneous resolution of symptoms. Khan et al. [40] also reported a low yield rate of 0.5% at a cost of approximately £11,000 per new diagnosis of MC, which is also reflected in our local data from the tertiary hospitals in Western Australia indicating low yield and yet a high cost.

As evident from the above, the incidence of MC is very low in the general population and is even lower in those >50 years who present with chronic watery diarrhoea. Hence, random colonic biopsies in the macroscopically normal colon should be targeted and reserved for patients with risk factors for MC. This will help reduce the healthcare cost burden and avoid unnecessary morbidity. Cotter et al. [31] created a scoring system to predict MC in patients with chronic diarrhoea. They reported that the model could predict 34.9% of their patient cohort being low risks, reducing the biopsy cost by 30%. This study’s population are screened by the family practitioner and booked directly for a colonoscopy; in future, a screening questionnaire as part of the referral would assist in decreasing the number of unnecessary colonoscopies.

Biopsy site and number 

In this study, most of our patients had varying numbers of biopsies taken from multiple locations. Interestingly, most of the MC-positive biopsies were from the left colon. This study also reported a positive correlation between the number of biopsies taken and the likelihood of having an MC-positive biopsy result. In a recent systematic review looking at determining the optimal sites and minimum biopsy number to diagnose MC, it concluded that a minimum of six biopsies should be taken from both the left and right sides of the colon. However, MC-positive biopsy seemed to be seen in most left-sided colon biopsies, which is congruent with this study’s findings [22]. This approach aligns with endorsed European Guidelines on MC that strongly recommends simultaneous biopsies from both left and right colonic sides because MC histological changes are demonstrated bilaterally in more than 95% of the cases [4]. Based on the findings of this study, as well as supporting literature, this paper’s recommendation would be to move away from random colonic biopsies but rather limit these biopsies to the right and left colonic sites and, better still, to only left side, while avoiding the rectum. This approach has been shown to have a high diagnostic yield and better cost savings.

Limitations 

Limitations of this paper include the retrospective design and the associated data collection issues; notably, there needed to be more complete data regarding why some patients who presented with chronic diarrhoea did not proceed to have biopsies at colonoscopy. If this is due to recent normal endoscopies, this paper may overestimate the diagnostic yield of colonoscopy in microcolitis patients. In addition, it was also limited by the fact that it did not evaluate the treatment and patient-reported outcomes of the patients diagnosed with microcolitis; this question is best suited for a future study designed and powered to answer such a question. The applicability of the result and recommendation based on this analysis will also differ in countries without or with a different approach to managing functional bowel issues. Nonetheless, this paper adds to the growing literature on the low diagnostic yield and yet high operating costs associated with colonoscopy and random biopsies. 

## Conclusions

The results and outcomes from this multi-centre study will add to the global understanding and debate on the yield rate of routine, random biopsies performed during colonoscopy to diagnose microscopic colitis as a cause of chronic diarrhoea. This is particularly important in this climate of fiscal responsibility and delivering a cost-conscious yet efficient and effective healthcare service that is both sustainable and responsive to community and individual needs. Random colonic biopsies for micro-colitis have a low yield (7.4%). They are, however, associated with clinically significant morbidity and healthcare costs. Positive biopsies were mostly from the left colon; it is time to rethink our practice of investigating microcolitis as a cause of chronic diarrhoea in patients > 50 years old. It may be targeted and limited to a specific population subset.
